# Sars-Cov-2 spike protein and plasma from COVID-19 patients induce extracellular traps by myeloid-derived suppressor cells

**DOI:** 10.3389/fcimb.2025.1612198

**Published:** 2025-11-13

**Authors:** Germana Grassi, Simona Gili, Rita Casetti, Zulema Antonia Percario, Nicola Tumino, Paola Vacca, Harpreet Kaur Lamsira, Roberta Nardacci, Stefania Notari, Veronica Bordoni, Eleonora Cimini, Flavia Cristofanelli, Dorotea Rubino, Francesca Nonini, Elisabetta Affabris, Luisa Marchioni, Chiara Agrati, Alessandra Sacchi

**Affiliations:** 1 Cellular Immunology and Pharmacology Laboratory, National Institute for Infectious Diseases Lazzaro Spallanzani, Istituto di Ricovero e Cura a Carattere Scientifico (IRCCS), Rome, Italy; 2 Molecular Virology and Antimicrobial Immunity Laboratory, Department of Science, Roma Tre University, Rome, Italy; 3 Immunology Research Area, Innate Lymphoid Cells Unit, Istituto di Ricovero e Cura a Carattere Scientifico (IRCCS), Bambino Gesù Children’s Hospital, Rome, Italy; 4 Departmental Faculty of Medicine, Saint Camillus International University of Health Sciences, Rome, Italy; 5 Oncoematologia e Officina Farmaceutica, Istituto di Ricovero e Cura a Carattere Scientifico (IRCCS), Bambino Gesù Children’s Hospital, Rome, Italy; 6 Clinical Division, National Institute for Infectious Diseases Lazzaro Spallanzani, Istituto di Ricovero e Cura a Carattere Scientifico (IRCCS), Rome, Italy

**Keywords:** SARS-CoV-2, MDSC, COVID-19, extracellular traps, spike

## Abstract

**Introduction:**

Polymorphonuclear-myeloid-derived suppressor cells (PMN-MDSC) are elevated in COVID-19 patients, playing a crucial role in suppressing the SARS-CoV-2 specific T-cell response and serving as an early marker for disease progression. In this study, we investigated the involvement of PMN-MDSC from COVID-19 patients in the formation of extracellular traps (ET).

**Methods:**

Fifty RT-PCR–confirmed severe COVID-19 patients admitted to the ICU and ten healthy donors were enrolled. PBMC were isolated from peripheral blood by density gradient centrifugation, and PMN-MDSC frequency was evaluated by flow cytometry. PMN-MDSC were isolated by immunomagnetic separation. ET extrusion was analyzed by immunofluorescence imaging. Apoptosis of pulmonary microvascular endothelial cells cultured with PMN-MDSC was measured by flow cytometry.

**Results:**

We found that platelet-rich plasma (PRP) from COVID-19 patients, unlike that from healthy donors, induced ET formation by PMN-MDSC. Furthermore, the PRP-induced ET was found to be independent of Toll-like receptor 4 (TLR4) signaling. Interestingly, the SARS-CoV-2 Spike protein itself can trigger ET formation via a TLR4-dependent pathway. Additionally, PMN-MDSC induced endothelial cell apoptosis through an ET-independent mechanism.

**Discussion:**

These findings highlight a previously unrecognized contribution of PMN-MDSCs to the thrombotic complications in severe COVID-19 cases, underscoring their detrimental impact on disease progression.

## 1 Introduction

Nearly 700 million people worldwide have been infected with the severe acute respiratory coronavirus 2 (SARS-CoV-2), the cause of coronavirus disease 2019 (COVID-19) (https://covid19.who.int/). About 7 million infected individuals died from infection.

SARS-CoV-2 replication induces cell damage and the release of pathogen-associated molecular patterns (PAMPs), stimulating a local inflammation characterized by increased secretion of the pro-inflammatory cytokines and chemokines (Huang et al., 2020). Beyond cytokine storm, lymphopenia, and increased neutrophil–lymphocyte ratios have been described in severe COVID-19 (Qin et al., 2020). However, the immunological mechanisms underlying the clinical presentation of SARS-CoV-2 infection and those influencing the disease outcome remain to be clearly defined.

Several reports have highlighted the expansion of myeloid-derived suppressor cells (MDSC) during COVID-19. (Grassi et al., 2022) MDSC are myeloid cells with suppressive functions and include two major subsets based on their phenotypic and morphological features: polymorphonuclear (PMN) and monocytic (M) MDSC. MDSC can inhibit T-cell proliferation (Nagaraj et al., 2007) (Rodriguez et al., 2007), suppress natural killer cell function (Li et al., 2009) and impair dendritic cell (DC) differentiation (Greifenberg et al., 2009) (Poschke et al., 2012).

During COVID-19, expanded MDSC can infiltrate the lung (Dean et al., 2021), inhibit the SARS-CoV-2-specific T cells (Sacchi et al., 2020), and induce platelet activation (Sacchi et al., 2021) thus contributing to the immune-mediated pathology of COVID-19. Indeed, some studies observed a higher frequency of MDSC in non-survival compared to survival COVID-19 patients (Jiménez-Cortegana et al., 2021) (Sacchi et al., 2020), suggesting that the frequency of MDSC could be used as a predictive marker of the disease outcome.

Beyond immune suppressive capacity, it has been demonstrated that PMN-MDSC from tumor-bearing mice were able to release extracellular traps (ET) similar to what neutrophils do (neutrophil extracellular traps, NET), interfering with cytotoxic activity towards cancer cells and promoting metastasis (Teijeira et al., 2020) (Ortiz-Espinosa et al., 2022).

NET release has been demonstrated to play a role in COVID-19 pathology. NETs consist of a diffuse, sticky web of extracellular DNA, and nuclear and granular proteins, and serve to ensnare and kill pathogens ^15^. In addition to catching pathogens, the cytotoxic molecules and proteases associated with NETs can potentially inflict significant tissue damage. Additionally, NET components have been suggested to be key activators of infection-induced coagulopathy (Kim and Jenne, 2016). Tracheal aspirates and pulmonary autopsies from COVID-19 patients showed NET-containing microthrombi and neutrophil infiltration (Middleton et al., 2020) (Veras et al., 2020). SARS-CoV-2 can directly induce the release of NETs by healthy neutrophils, and NETs released by SARS-CoV-2-activated neutrophils promote lung epithelial cell death *in vitro* (Veras et al., 2020).

The original conventional characterization of MDSCs as immature myeloid progenitors arrested in their differentiation has been refined by emerging evidence indicating that both M-MDSC and PMN-MDSC subsets may also derive from more differentiated monocytes and granulocytes that have acquired immunoregulatory functions (Millrud et al., 2017). PMN-MDSC are very similar to neutrophils, but whether they retain some of the neutrophil functions is unclear.

To date, the capacity of PMN-MDSCs to form ET in the context of severe COVID-19 remains poorly characterized. This study aimed to assess whether PMN-MDSCs from patients with severe COVID-19 retain the capacity to form ET.

We found that PMN-MDSC from COVID-19 patients were able to release ET upon stimulation with autologous plasma or with SARS-CoV-2 spike protein, shedding light on previously unrecognized aspects of their immunosuppressive or pro-inflammatory activity.

## 2 Materials and methods

### 2.1 Study population

SARS-CoV-2 infected patients (n = 50), confirmed by RT-PCR, were enrolled at the National Institute for Infectious Diseases (INMI) “Lazzaro Spallanzani” (Rome, Italy). Patients’ characteristics are summarized in **Table 1**. All patients had severe COVID-19 disease and required intensive care unit admission (ICU). Seventy-eight percent of patients presented one or more bacterial or fungal co-infections such as Acinetobacter spp, Klebsiella spp, Pseudomonas spp, Staphylococcus spp, Streptococcus spp, Aspergillus spp, Candida spp, and others. Sixty-four percent of co-infected patients had bacteremia. Among the patients with coinfections, 15 were infected with both Gram-positive and Gram-negative bacteria, 13 with Gram-negative bacteria, and 11 with Gram-positive bacteria. All co-infected patients received antibiotic or antimycotic treatment, and 2 were also treated with remdesivir. Among the subjects without co-infection, two received an antibiotic treatment. Eighty-four percent had other co-morbidities, such as obesity, diabetes, and cardiovascular diseases. The 36% received anti-COVID-19 vaccination. The presence of any tumor type represented an exclusion criterion. Signed written informed consent was obtained from all patients. Healthy individuals (HD, n=10) were included as controls. The study was approved by the Institutional Review Board of the INMI (approval number: 9/2020). Granulocyte-colony stimulating factor (G-CSF) mobilized donors were enrolled at Bambino Gesù Children’s Hospital, Rome, Italy. They received subcutaneous administration of G-CSF for five days (until apheresis) at a dose of 10− 12 μg/kg/day. Informed consent was obtained by G-CSF mobilized healthy donors to participate in this study, which was approved by the Bambino Gesù Children’s Hospital (Rome, Italy) ethics committees (Prot. n. 132 28/01/2019).

**Table 1:** Patient's characteristics (n=50).

Sex (Female)	Age	Time from SarsCoV-2 diagnosis	Co-infections	Bacteriemia	Co-morbidity	OTI
n=19 (38%)	median: 67,5 years range: 27–88 years	median: 10days range: 5–90 days	n=39 (78%); female n=16	n=25 (64,1% of coinfected)	n=42 (84%); female n=15	n= 46 (92%); female n=18

OTI, orotracheal intubation.

### 2.2 Plasma samples preparation

Platelet-rich (PRP) was obtained from heparin-treated whole blood by centrifugation for 10 min at 1100 rpm, at 4°C. was maintained on ice until use.

### 2.3 Peripheral blood mononuclear cells and PMN-MDSC isolation

Ten ml of heparin anti-coagulated whole blood samples were obtained from patients. Density gradient centrifugation (Lympholyte-H, Cederlane, Burlington, USA) was used to isolate peripheral blood mononuclear cells (PBMC). PBMC were resuspended in RPMI 1640 (Corning Incorporated, New York, USA) supplemented with 10% heat-inactivated fetal bovine serum (FBS) (EuroClone, Milan, Italy), 2 mmol/L L-glutamine (Corning Incorporated, New York, USA), penicillin/streptomycin solutions, 100X (Corning Incorporated, New York, USA) and 10 mmol/L HEPES buffer (N-2- hydroxyethylpiperazine-N-2-ethane sulfonic acid, Gibco, USA). PMN-MDSC were isolated from PBMC from COVID-19 patients by magnetic cell isolation technology. Positive selection was performed using CD15+ microbeads following manufacturer’s instruction (Miltenyi Biotec, Bergisch Gladbach, Germany). PMN-MDSC purity was >95% as verified by flow cytometry (data not shown). PMN-MDSC viability was evaluated by trypan blue exclusion. PBMC were also obtained from G-CSF mobilized healthy donors after density gradient centrifugation (Lympholyte-H, Cederlane, Burlington, USA). PMN-MDSC were isolated from PBMC of mobilized donors using CD66b+ microbeads following manufacturer’s instruction (Miltenyi Biotec, Bergisch Gladbach, Germany). PMN-MDSC purity was >98% as verified by flow cytometry (data not shown).

### 2.4 Flow cytometry

MDSC frequency was evaluated by flow cytometry by staining PBMC using dry pre-formulated antibody panels DuraClone (CD11b-FITC, DRAQ7, HLA-DR-ECD, CD45-KrO, CD14-PC5.5, CD33-PC7, CD80-APC, CD19-APC-alexa750, CD56-APC-alexa750, CD3-APC-alexa750, CD15-Pacific-Blue (Beckman Coulter, Brea,CA,USA) following manufacturer’s procedures. Briefly, 2-5x10^5 PBMC were washed and resuspended in PBS (100 uL) and transferred to the duraclone tube. After 15 min. PBMC were washed and resuspended in 1% paraformaldehyde (PFA). TLR4 expression levels on PMN-MDSC were evaluated by staining PBMCs with different mixtures of monoclonal antibodies: anti-CD45 KrO, anti-CD15 FITC and anti-TLR4 PE (Miltenyi Biotec, Bergisch Gladbach, Germany). After 15 min of incubation, the cells were washed with PBS and fixed with 1% PFA. Acquisition of 100,000 events was performed in the leukocyte-gated population on Cytoflex LX cytometer (Beckman Coulter, Brea, CA, USA) and analyzed by CytExpert software (Beckman Coulter, Brea, CA, USA).

### 2.5 Immunofluorescence and confocal imaging

Purified PMN-MDSC (3,5x10^5^) were seeded in poly-D-lysine coated 4-well Chamber Slide (Permanox Slide, Thermo Fisher Scientific, Rochester, New York, NY, USA) in RPMI 1640 supplemented with 2 mmol/L L-glutamine, penicillin/streptomycin solutions, 100X, and 10 mmol/L HEPES buffer.

Cells were stimulated with 10% PRP from patients or HD or recombinant SARS-CoV-2 Spike active trimer (200ng/ml, R&D Systems, USA). Where indicated the TLR4 inhibitor TAK-242 (0.1μM) (Calbiochem) was added 30 minutes before plasma or SARS-CoV-2 Spike protein stimulation. Cells were cultured at 37°C in humidified air with 5% CO_2_. After two hours, PMN-MDSC were washed with Hanks’Balanced Salt Solution, and Syto Green solution (25 nM, Life Technologies) was added to stain dsDNA.

After fixation with 4% paraformaldehyde (Sigma-Aldrich, P6148) for 20 min at room temperature the slides were mounted using the ProLong Gold antifade reagent with DAPI (Molecular Probes, Invitrogene, USA).

Image acquisition was carried out using a Leica TCS SP2 confocal microscopy (Leica microsystems). Image processing and analysis were done using Leica Confocal Software (LCS).

### 2.6 Extracellular DNA quantification

Purified PMN-MDSC (2x10^5^ cells/well) were cultured in 96-well flat plates (Corning-Incorporated, New York, NJ, USA), in the above-described medium. Cells were stimulated as mentioned above for 2h at 37°C in humidified air with 5% CO^2^. Extracellular DNA was detected by Pico488 dsDNA quantification kit (Lumiprobe, USA) following the manufacturer’s procedure. Briefly, samples were diluted with the buffer and an equal volume of the Pico488 dye working solution. The final DNA concentration was acquired and analyzed by “Magellan Pro V 7.5” (Tecan Trading AG, Switzerland).

### 2.7 Western blot assay

Western blot analyses on cell lysates were performed by washing cells twice with ice-cold PBS (pH 7.4) and lysing them for 30 min on ice with lysis buffer (50 mM Tris pH 7.4, 150 mM NaCl, 0,25% sodium deoxycholate, 1 mM EDTA, 1 mM EGTA, 1% Triton X-100, 0.5% non-ionic detergent IGEPAL CA-630 (Sigma-Aldrich, Milan, Italy), 1 mM sodium orthovanadate, 20 mM sodium fluoride, 1 µg/mL leupeptin and pepstatin A, 2 μg/mL aprotinin and 1 mM phenylmethylsulfonyl fluoride (PMSF). Whole-cell lysates were centrifuged at 6000× *g* for 10 min at 4°C. The protein concentration of cell extracts was determined by protein assay dye reagent (Bio-Rad Laboratories). Aliquots of cell extracts containing 30 µg of total proteins were resolved by 8% sodium dodecyl sulphate-polyacrylamide gel electrophoresis (SDS-PAGE) and transferred by electroblotting on 0.45 µm pore size nitrocellulose membranes (Amersham™, Merck Life Science S.r.l., Italy). For the immunoassays, membranes were blocked in 3% bovine serum albumin (BSA) fraction V (Biofroxx, Einhausen, Germany) in TTBS/EDTA (10 mM Tris pH 7.4, 100 mM NaCl and 1 mM EDTA, 0.1% Tween 20) for 30 min at room temperature (RT) and then incubated for 1h at RT or overnight at 4°C with specific primary antibodies diluted in 1% BSA/TTBS-EDTA. The antibodies used in immunoblotting were the following: rabbit anti-human ACE2 (Life Span BioSciences), mouse anti-human β-Actin (Santa Cruz Biotechnology), goat Anti-Rabbit antibody (H + L)-HRP conjugate (Bio-Rad Laboratories), and goat anti-mouse antibody (Enzo Life Technologies, Farmingdale, NY, USA).

The immune complexes were detected by enhanced chemiluminescence reaction (ECL Fast Pico; Immunological Sciences, Rome, Italy) and the ChemiDoc XRS (Bio-Rad, Hercules, CA, USA) instrument and the Image Lab software (Bio-Rad) were used to reveal the chemiluminescence signal.

### 2.8 PMN-MDSC culture with endothelial cells

Human Pulmonary Microvascular Endothelial Cells were seeded at 2x10^4^/cm^2^ (ECs, PromoCell Heidelberg, Germany) and cultured in Endothelial Cell Growth medium MV (PromoCell, Heidelberg, Germany), supplemented with SupplementMix (PromoCell, Heidelberg, Germany) in a humidified atmosphere (5% CO_2_) at 37°C. The medium was refreshed every 3 days. Once confluence was reached, cells were detached using the Animal Component-Free (ACF) Cell Dissociation Kit (STEMCELL Vancouver, BC, Canada) and seeded on 24-well plates (Corning Incorporated, New York, NY, USA) at a density of 1.1 × 10^5^ cells/mL. When confluence was reached, purified PMN-MDSC were added (3× 10^5^/mL) and cells were stimulated with recombinant SARS-CoV-2 Spike active trimer (200ng/ml, R&D Systems, USA) for 24 hours. ECs viability was evaluated by flow cytometry (Annexin V Apoptosis Detection Kit, eBioscience™).

### 2.9 Statistical analysis

GraphPad Prism version 9.3.1 for Windows (GraphPad Software) was used to perform statistical analyses. The non-parametric Friedman test with Dunn’s correction or the Mann-Whitney test were used to compare continuous variables. The p-value < 0.05 was considered statistically significant.

## 3 Results

### 3.1 The PMN-MDSC frequency was not associated with bacterial co-infections

**Figure 1:**
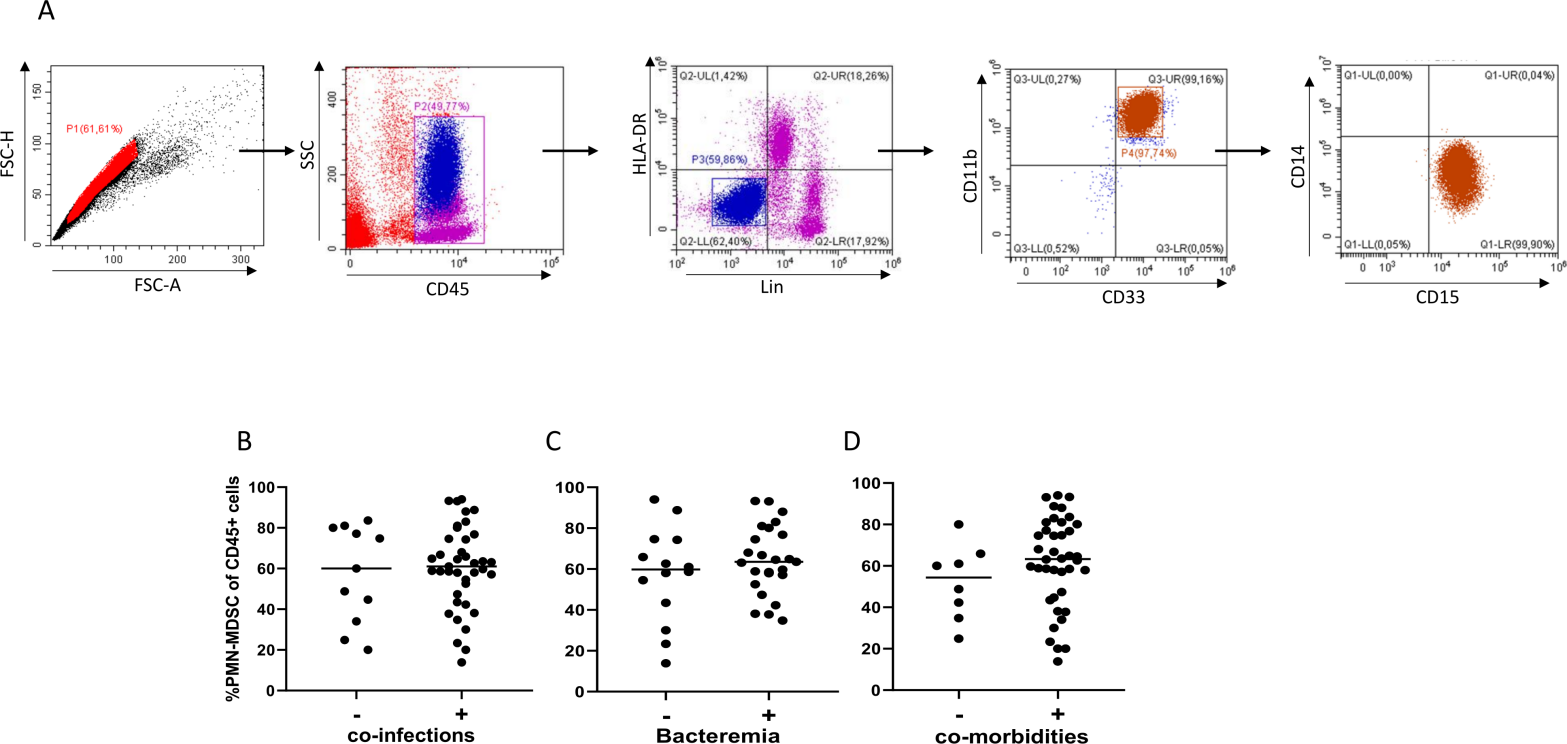
PMN-MDSC from severe COVID-19 patients are not affected by co-infections. **(A)** Representative plots of the adopted gating strategy to identify MDSC. Doublets were excluded in the FSC-H/FSC-A dot plot. In the immunological plot (Side Scatter (SSC)/CD45), the CD45+ cells were gated followed by gating on Lin-(CD3-CD19-CD56-)/HLA-DRlow/- cell. In this gate, we selected the CD33+/CD11b+ cells, and among these, we evaluated the expression of CD14 and CD15. Since 100% of HLA-DR-/Lin-/CD33+/CD11b+ cells were CD14-/CD15+ (PMN-MDSC), PMN-MDSC frequency was calculated as the percentage of CD15+ cells in the CD45+ cells gate. Frequency of PMN-MDSC in patients with and without co-infection (n=39, n=11 respectively) **(B)**, with and without bacteremia (n=25, n=14 respectively) **(C)**, with and without other co-morbidities (n=40, n=10 respectively) **(D)**. Results are shown as scatter dot plot and median. The Mann-Whitney test was applied. P<0.05 was considered statistically significant.

Most of the enrolled patients presented bacterial co-infection at the time of enrolment (39 out of 50, 78%), and we wondered whether co-infection could affect PMN-MDSC frequency. **Figure 1A** shows the gating strategy used to identify circulating MDSC among PBMC. We found that PMN-MDSC frequency was comparable between COVID-19 patients with and without bacterial co-infections (**Figure 1B**). Moreover, among co-infected patients, we did not find any difference in the PMN-MDSC frequency between patients with and without bacteremia (**Figure 1C**). These data indicate that, in COVID-19 patients, the presence of bacterial co-infections did not influence PMN-MDSC frequency, suggesting that the main driver of PMN-MDSC expansion was the SARS-CoV-2 infection. Accordingly, the percentage of PMN-MDSC from patients with other co-morbidities did not differ from those without (**Figure 1D**). We did not find any difference in the PMN-MDSC percentage between males and females (data not shown).

### 3.2 PMN-MDSC from patients with COVID-19 extrude ET upon plasma stimulation

**Figure 2:**
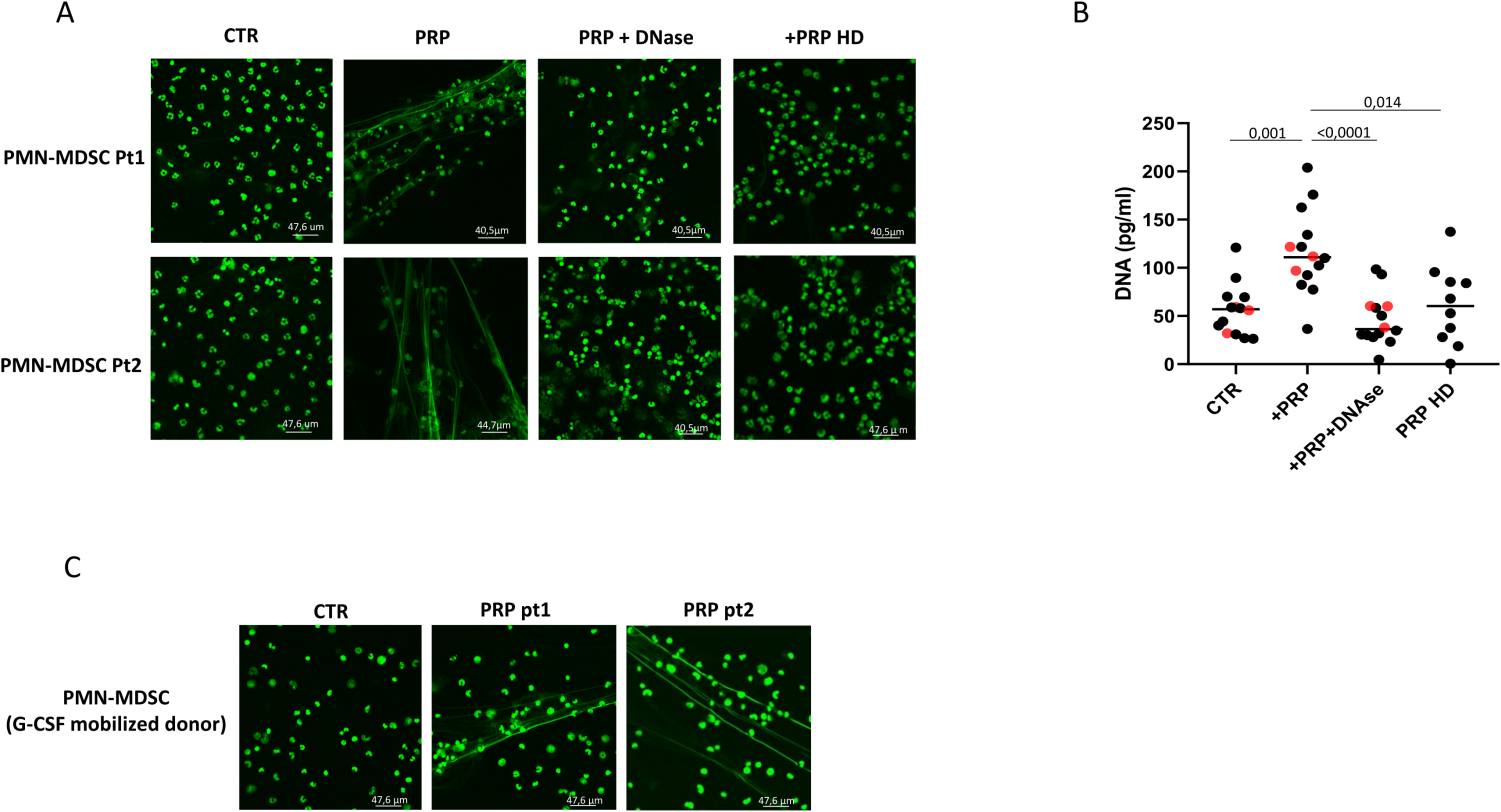
Plasma from COVID-19 patients induces ET release by PMN-MDSC. **(A)** Confocal microscopy images of PMN-MDSC from two representative COVID-19 patients and HD not treated (CTR) and treated with platelet-rich plasma (PRP). **(B)** DNA quantification on culture supernatants of PMN-MDSC from COVID-19 patients (n=16, 13 out of 16 with co-infections) stimulated for 2 hours with PRP, PRP and DNAse, and PRP from healthy donors (n=9, PRP HD). Results are shown as scatter dot plot and median. Friedman test with Dunn’s correction was applied. **(C)** Confocal microscopy images of PMN-MDSC isolated from one representative G-CSF mobilized healthy donor treated with PRP from two COVID-19 patients.

It has been shown that plasma from COVID-19 patients can induce NET by neutrophils, (Middleton et al., 2020) (Kelly et al., 2023) We then evaluated whether PMN-MDSC from COVID-19 patients can release ET upon plasma stimulation. To this aim, purified PMN-MDSC from 16 COVID-19 patients (13 out of 16 with co-infections) were stimulated with 10% of autologous platelets enriched plasma (PRP), and after 2 hours, the release of ET was analyzed by confocal microscopy. We found that PMN-MDSC were able to produce well-organized DNA filaments suggestive of ET upon PRP stimulation (**Figure 2A**); DNA release was confirmed by treating with DNAse after which DNA filaments were degraded (**Figure 2A**). Differently, stimulation with PRP from HD was not able to induce ET by PMN-MDSC. In the same experimental conditions, the amount of free DNA in the culture supernatants was quantified by pico488 fluorescence. **Figure 2B** confirms the results obtained by confocal microscopy, showing an increase in free DNA after stimulation with PRP from COVID-19 patients but not from HD. Red points indicate DNA release from PMN-MDSC isolated from non-co-infected patients. This result suggests that bacterial or fungal co-infection has no major role in inducing ET production.

We then wondered if the capacity to release ET was a peculiar feature of PMN-MDSC from COVID-19 patients. It has been demonstrated that G-CSF stem cell mobilization in human donors induces PMN-MDSC (Luyckx et al., 2012). Mature CD10+ and immature CD10- neutrophils present in G-CSF-treated donors display opposite effects on T cells (Marini et al., 2017; Tumino et al., 2020). Thus, purified PMN-MDSC from G-CSF-mobilized healthy individuals were treated with PRP from patients with COVID-19. **Figure 2D** shows that PMN-MDSC from HD extrude ET when stimulated with PRP of infected patients, indicating that ET production is not a peculiar feature of PMN-MDSC from SARS-CoV-2 infected patients. To confirm that PRP from COVID-19 patients induced ET formation, PMN-MDSC from G-CSF-mobilized healthy individuals were stimulated with PRP from COVID-19 patients and after 2 hours, the elastase activity associated with ET was evaluated by NETosis assay kit. **Supplementary Figure 1** shows that PRP from COVID-19 patients induced the release of ET-associated elastase, confirming the formation of ET by PMN-MDSCs.

### 3.3 Plasma-induced ET release by PMN-MDSC is TLR4-independent

**Figure 3:**
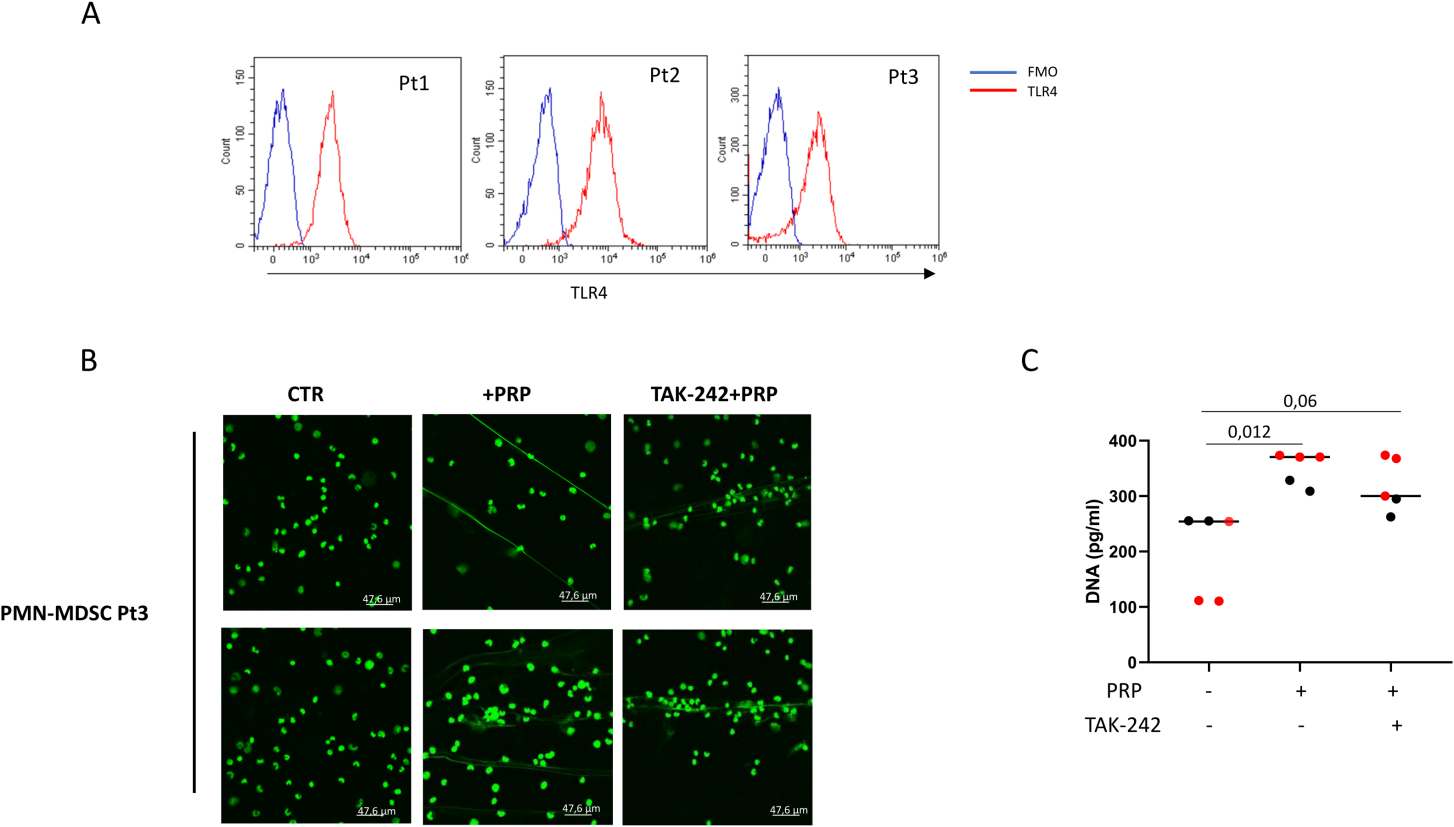
PRP-induced ET release by PMN-MDSC is independent of TLR4. PMN-MDSC were identified as shown in **Figure 1A**. In the gate of CD15+ cells the expression of TLR4 was evaluated. **(A)** TLR4 expression on CD15+ cells from 3 representative patients. **(B)** Confocal microscopy images of purified PMN-MDSC treated with PRP, PRP and a TLR4 inhibitor (TAK-242) from one representative COVID-19 patient (two fields per condition are shown). **(C)** DNA quantification on culture supernatants of PMN-MDSC from COVID-19 patients (n=5, with bacterial co-infections, 3 with Gram neg. e 2 with Gram pos. bacteria) stimulated for 2 hours with PRP, and PRP and a TLR4 inhibitor (TAK-242). Results are shown as scatter dot plot and median. Friedman test with Dunn’s correction was applied. P<0.05 was considered statistically significant.

In neutrophils, a TLR4-dependent NET formation has been described (Zhang et al., 2021). We wondered whether PRP-induced ET released by PMN-MDSC was TLR-4-mediated. First, we evaluated whether PMN-MDSC from COVID-19 patients express TLR4 and found that this receptor is present on the PMN-MDSC surface (**Figure 3A**). We then analyzed whether TLR4 inhibition could block ET release by PMN-MDSC. TLR4 inhibition did not affect ET extrusion by PMN-MDSC upon PRP stimulation (**Figures 3B, C**), indicating that the mechanism driving PMN-MDSC ET release was TLR4-independent.

### 3.4 SARS-CoV-2 spike protein induces ET by PMN-MDSC through TLR4

**Figure 4:**
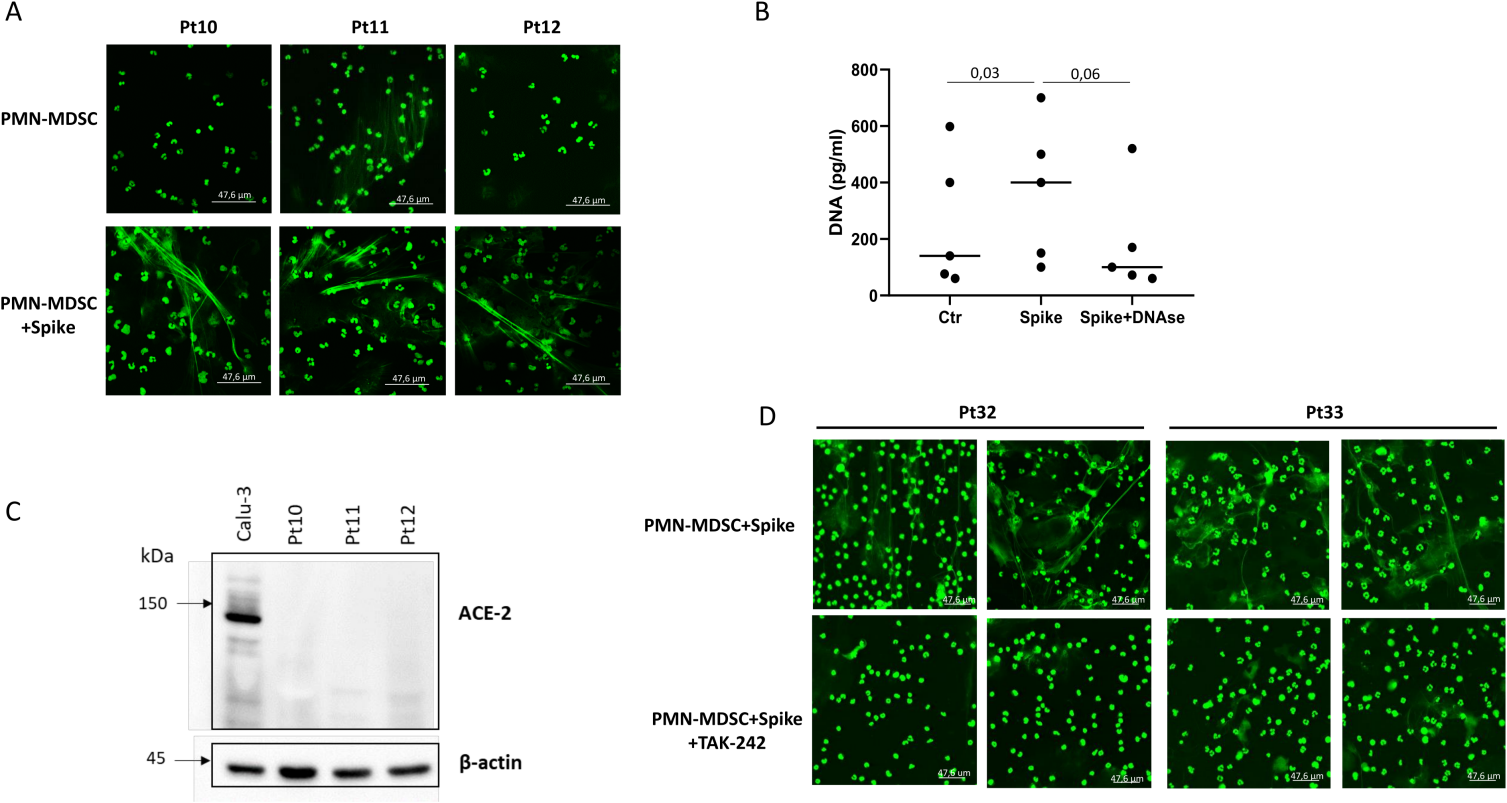
SARS-CoV-2 Spike protein induces ET extrusion by PMN-MDSC in a TLR4-dependent mechanism. **(A)** Confocal microscopy images of PMN-MDSC from 3 representative patients treated with Spike. **(B)** DNA quantification on culture supernatants of PMN-MDSC from COVID-19 patients (n=5, with co-infections) stimulated for 2 hours with Spike protein. Results are shown as scatter dot plot and median. Friedman test with Dunn’s correction was applied. P<0.05 was considered statistically significant. **(C)** ACE-2 expression in purified PMN-MDSC from 3 representative COVID-19 patients out of 5 tested (all with co-infections). β-actin has been used as an internal control for the loaded samples. **(D)** Confocal microscopy images of PMN-MDSC from two representative COVID-19 patients treated with Spike protein, or Spike and a TLR4 inhibitor (TAK-242).

It has been demonstrated that SARS-CoV-2 Spike protein could be involved in NET extrusion (Youn et al., 2021). We thus evaluated whether the Spike protein could stimulate ET by PMN-MDSC. ET production by purified PMN-MDSC was observed upon Spike stimulation (**Figures 4A, B**), which was confirmed by DNAse treatment. The release of ET was further confirmed by analyzing the ET-associated elastase using the NETosis assay kit (**Supplementary Figure 1**). It has been reported that neutrophils express ACE2 and an ACE2-dependent release of NET by neutrophils has been shown during SARS-CoV-2 infection (Veras et al., 2020). We found that PMN-MDSC from COVID-19 patients did not express ACE2 (**Figure 4C**), excluding the ACE2-mediated ET extrusion. However, Spike protein can bind to other receptors such as TLR4 (Zhao et al., 2021). We then evaluated whether Spike-induced ET by PMN-MDSC was mediated by TLR4. We found that the TLR4 inhibitor TAK-242 inhibited ET release by spike-stimulated PMN-MDSC (**Figure 4D**), indicating a TLR4-dependent mechanism.

### 3.5 PMN-MDSC from SARS-CoV-2 infected patients induce endothelial cell damage

**Figure 5:**
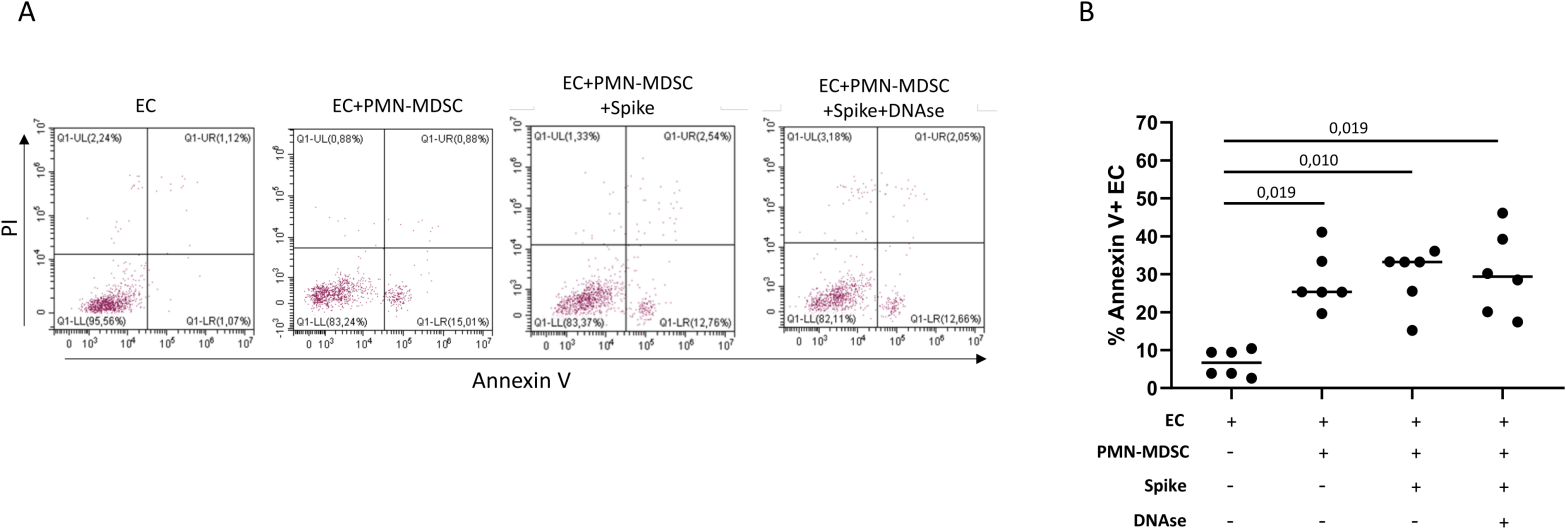
PMN-MDSC induce endothelial cell apoptosis independently from ET release. **(A)** Representative flow cytometry plots of Annexin V and propidium iodate (PI) expression in EC after 24h of culture with purified PMN-MDSC (1:2 ratio) from one COVID-19 patient treated or not with Spike protein, and Spike and DNAse. **(B)** Cumulative data of the percentage of Annexin V+ EC after culture with purified PMN-MDSC from COVID-19 patients (n=6, with co-infections), treated or not with Spike protein, and Spike and DNAse. Results are shown as scatter dot plot and median. Friedman test with Dunn’s correction was applied. P<0.05 was considered statistically significant.

Neutrophils and NET can damage endothelial cells and increase endothelial permeability (Ma et al., 2019). Since the capacity of PMN-MDSC to release ET, we evaluated whether MDSC from COVID-19 patients can induce endothelial cell dysfunction. To this aim, primary human microvascular endothelial cells (EC) were cultured with purified PMN-MDSC from COVID-19 patients and treated with Spike protein to induce ET formation. After 24h, we evaluated EC apoptosis by flow cytometry. **Figure 5A** shows the EC expression of Annexin V and PI after culture with PMN-MDSC in the indicated conditions. We observed that PMN-MDSC alone were able to induce EC apoptosis, in particular, early apoptosis, being Annexin V+ PI- (**Figure 5A**). Stimulation with the Spike protein did not affect EC viability compared with PMN-MDSC alone, suggesting that ET are not involved in the PMN-MDSC-induced EC death. Indeed, the treatment with DNAse did not affect the capacity of PMN-MDSC to induce EC death (**Figures 5A, B**), confirming that PMN-MDSC-induced EC apoptosis was not mediated by ET release.

## 4 Discussion

Severe acute respiratory syndrome-coronavirus (SARS-CoV-2) is the causative agent of the coronavirus disease 2019 (COVID-19). The pathogenesis of SARS-CoV-2 initiates when the viral particles invade airway epithelial cells, alveolar epithelial cells, vascular endothelial cells, and macrophages in the lung (Walls et al., 2020). Viral replication induces cell death and the release of pathogen-associated molecular patterns (PAMPs), that induce local inflammation, mediated by the secretion of the pro-inflammatory cytokines and chemokines (Huang et al., 2020). These cytokines and chemokines attract monocytes and T lymphocytes from the blood into the infected lung (Xu et al., 2020). In some cases, a dysfunctional immune response occurs, which triggers a cytokine storm that mediates systemic inflammation (Zhou et al., 2020).

Thanks to their immunosuppressive capabilities, MDSC could play a crucial role in limiting excessive inflammation or inflammatory storm of COVID-19 (Perfilyeva et al., 2022); however, excessive inflammation or inflammatory storms lead to accumulation of MDSC in the peripheral blood of COVID-19 patients that participate in the pathological process of the disease rather than providing benefits (Falck-Jones et al., 2021) (Agrati et al., 2020).

PMN-MDSC share several features with neutrophils. Indeed, in cancer models, PMN-MDSC were able to extrude ET contributing to disease progression (Teijeira et al., 2020) (Ortiz-Espinosa et al., 2022). The pathological role of neutrophils through NET production during COVID-19 has been depicted (Veras et al., 2020) (Lebourgeois et al., 2022).

In this study, we evaluated the capacity of PMN-MDSC from severe COVID-19 patients to produce ET. PMN-MDSC were identified by flowcytometry as CD45+ Lin-HLADR- CD11b+ CD14-CD15+CD33+ cells that we have previously demonstrated to have suppressive functions ^10,31^.

The COVID-19 patients enrolled in this study had several bacterial co-infections. Thus, we first evaluated the effect of bacterial co-infections on PMN-MDSC frequency in COVID-19 patients receiving intensive care and found no impact; moreover, co-morbidity other than infections and anti-COVID-19 vaccination did not affect the PMN-MDSC percentage, indicating that PMN-MDSC expansion is mainly driven by SARS-CoV-2 infection-induced disease.

Herein, we demonstrated that PMN-MDSC from patients with severe COVID-19 were able to produce ET when stimulated with autologous plasma. The capacity to produce ET was not restricted to PMN-MDSC from infected patients; indeed, PMN-MDSC from G-CSF mobilized healthy individuals were able to produce ET when stimulated by COVID-19 PRP, indicating that PRP from COVID-19 patients delivers signals inducing ET formation by PMN-MDSC from both COVID-19 patients and HD. Although two different markers were used for the purification of MDSCs from patients and healthy donors (CD15 and CD66, respectively), PMN-MDSCs express both markers (Brandau et al., 2013) (Bronte et al., 2016), thereby supporting the conclusion that the same cellular populations were isolated. However, we cannot entirely exclude the possibility of subtle differences between these populations.

Several stimuli can induce NET formation by neutrophils, such as lipopolysaccharide (LPS) and High Mobility Group Box 1 (HMGB1) by stimulating TLR4 (Thomas and Schroder, 2013) (Huang et al., 2015). Our data indicate that, despite PMN-MDSC express TLR4, PRP-induced ET was TLR4-independent, excluding that HMGB1 or LPS could be involved in PRP-induced ET by PMN-MDSC. The capacity of plasma from patients without co-infections to induce the formation of ET further excludes a bacterial/fungal product involvement.

Recently, it has been shown that viable SARS-CoV-2 can directly induce the release of NET by healthy neutrophils. Mechanistically, NET triggered by SARS-CoV-2 depends on angiotensin-converting enzyme 2 (ACE-2) and serine protease TMPRSS2, and active viral infection and/or replication is necessary to induce NET release (Veras et al., 2020). We found that, unlike neutrophils, PMN-MDSC do not express ACE-2, excluding that the spike protein or viral particles potentially present in the plasma can act through ACE-2. Other factors could induce NET; it has been described that patient-derived SARSCoV-2 spike-antibody immunocomplexes (mainly IgA) from multisystem inflammatory syndrome in children (MIS-C) can trigger NETosis in healthy donor neutrophils (Boribong et al., 2022). Increased levels of IgA in circulation have been associated with severe COVID-19 in adults (Hasan Ali et al., 2021). Furthermore, IgA can be a potent inducer and potentiator of NET formation (Aleyd et al., 2014). Extracellular vesicles (EVs) from the plasma of COVID-19 patients have been shown to contribute to neutrophil extracellular trap formation through mechanisms independent of ACE2 and TLR4 (Liao et al., 2025). Notably, CLEC5A has been identified as a key mediator in this process (Sung et al., 2022). Whether immune complexes and EV may have a role in the PRP-induced ET production by PMN-MDSC remains to be established.

SARS-CoV-2 spike protein may bind and activate TLR4 (Zhao et al., 2021). We found that PMN-MDSC express TLR4 and were able to release ET upon stimulation with recombinant trimeric spike protein in a TLR4-dependent manner. To our knowledge, this is the first observation of a Spike-induced TLR4-dependent ET release by PMN-MDSC. Indeed, it has been proposed that spike protein can induce NET by neutrophils by interacting with C-type lectin receptors but not with TLR4 (Youn et al., 2021). These data suggest that SARS-CoV-2 may induce ET by neutrophils and PMN-MDSC through different mechanisms.

We also found that PMN-MDSC from COVID-19 patients induced endothelial cell death independently of ET release, suggesting other mechanisms through which MDSC may contribute to COVID-19 immunopathogenesis.

NET formation traps microorganisms to inhibit their spread. However, in excess, these traps can also cause prothrombotic events (von Brühl et al., 2012). Our results indicate that PMN-MDSC are involved in the pathogenesis of COVID-19 not only by impairing adaptive immune response; indeed, the Spike-TLR4-induced ET could contribute to the downstream inflammatory pathways, damaging pulmonary epithelial cells. Moreover, ET may interact with platelets to activate a thrombo-inflammation cascade, which promotes lung damage in COVID-19. The formation of ET can also lead to the production of excessive cytokines and chemokines, such as IL1β, IL6, IL8, and TNF-α, which may contribute to the cytokine storm leading to ARDS, and death.

Weiqi and co-authors suggest that SARS-CoV-2 could hijack histones from neutrophil NETosis to promote its host cell attachment and entry process (Hong et al., 2022), thus enhancing viral spread. In a cancer model, it has been shown that NET released by neutrophils and MDSC could cloak tumor cells to the immune system (Teijeira et al., 2020). Whether ET release by PMN-MDSC from COVID-19 may mask SARS-CoV2 infected cells to the immune system or may support/inhibit viral spread needs to be investigated.

Since validated markers for PMN-MDSC are still lacking, the potential contamination of low-density neutrophils in purified PMN-MDSC represents a limitation of this study and, more broadly, of all MDSC studies.

A second limitation of this study may reside in the simplified culture model, which consists of purified PMN-MDSC cultured with plasma or Spike protein, without considering the complex interplay *in vivo* with other cells or factors that could limit or enhance ET formation. However, our data highlight the potential capability of PMN-MDSC to extrude ET under appropriate stimuli.

We acknowledge that the number of patients included in this study is limited; however, the consistency and reproducibility of the results across independent donors, along with the clear mechanistic involvement of TLR4 in Spike-induced ET by PMN-MDSC, support the robustness and biological relevance of our findings. However, a confirmation in larger cohorts will be essential to further validate these observations.

In conclusion, our findings demonstrate that PMN-MDSCs are capable of producing ETs upon stimulation with PRP from COVID-19 patients or with the SARS-CoV-2 Spike protein, through TLR4-independent and TLR4-dependent mechanisms, respectively. These results suggest a previously unrecognized contribution of PMN-MDSCs to the thromboinflammatory processes characteristic of severe COVID-19. By elucidating a novel functional aspect of PMN-MDSCs, our study provides important insights into the immunopathology of SARS-CoV-2 infection and may help uncover new mechanisms of disease progression, with potential implications for the development of targeted therapeutic strategies.

## Data Availability

The raw data supporting the conclusions of this article will be made available by the authors, without undue reservation.
