# Correction: Sars-Cov-2 spike protein and plasma from COVID-19 patients induce extracellular traps by myeloid-derived suppressor cells

**DOI:** 10.3389/fcimb.2026.1810696

**Published:** 2026-02-26

**Authors:** Germana Grassi, Simona Gili, Rita Casetti, Zulema Antonia Percario, Nicola Tumino, Paola Vacca, Harpreet Kaur Lamsira, Roberta Nardacci, Stefania Notari, Veronica Bordoni, Eleonora Cimini, Flavia Cristofanelli, Dorotea Rubino, Francesca Nonini, Elisabetta Affabris, Luisa Marchioni, Chiara Agrati, Alessandra Sacchi

**Affiliations:** 1 Cellular Immunology and Pharmacology Laboratory, National Institute for Infectious Diseases Lazzaro Spallanzani, Istituto di Ricovero e Cura a Carattere Scientifico (IRCCS), Rome, Italy; 2 Molecular Virology and Antimicrobial Immunity Laboratory, Department of Science, Roma Tre University, Rome, Italy; 3 Immunology Research Area, Innate Lymphoid Cells Unit, Istituto di Ricovero e Cura a Carattere Scientifico (IRCCS), Bambino Gesù Children’s Hospital, Rome, Italy; 4 Departmental Faculty of Medicine, Saint Camillus International University of Health Sciences, Rome, Italy; 5 Oncoematologia e Officina Farmaceutica, Istituto di Ricovero e Cura a Carattere Scientifico (IRCCS), Bambino Gesù Children’s Hospital, Rome, Italy; 6 Clinical Division, National Institute for Infectious Diseases Lazzaro Spallanzani, Istituto di Ricovero e Cura a Carattere Scientifico (IRCCS), Rome, Italy

**Keywords:** SARS-CoV-2, MDSC, COVID-19, extracellular traps, spike

Author “Chiara Agrati” was erroneously assigned to affiliation “Departmental Faculty of Medicine, Saint Camillus International University of Health Sciences, Rome, Italy”. This affiliation has now been removed for author “Chiara Agrati” and the correct affiliation was assigned.

The original version of this article has been updated.

