# Supplementary figures and images for: Sars-Cov-2 spike protein and plasma from COVID-19 patients induce extracellular traps by myeloid-derived suppressor cells
Source: Front Cell Infect Microbiol. 2025 Nov 13;15:1612198. doi: 10.3389/fcimb.2025.1612198 (PMC12657375; doi:10.3389/fcimb.2025.1612198)

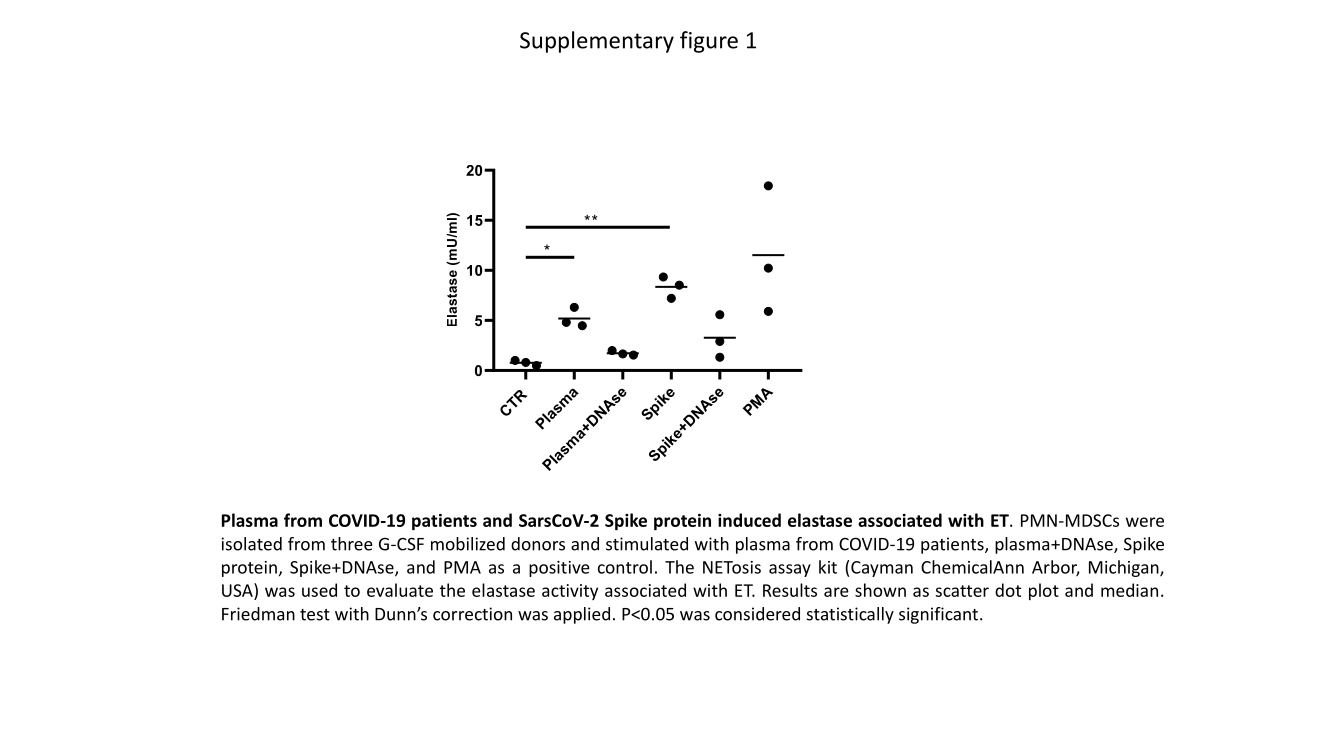

Supplement: Supplementary file 1 [file Image1.tif]
